# Evidence for disrupted copper availability in human spinal cord supports Cu^II^(atsm) as a treatment option for sporadic cases of ALS

**DOI:** 10.1038/s41598-024-55832-w

**Published:** 2024-03-11

**Authors:** James B. W. Hilton, Kai Kysenius, Jeffrey R. Liddell, Stephen W. Mercer, Bence Paul, Joseph S. Beckman, Catriona A. McLean, Anthony R. White, Paul S. Donnelly, Ashley I. Bush, Dominic J. Hare, Blaine R. Roberts, Peter J. Crouch

**Affiliations:** 1 Department of Anatomy and Physiology, The University of Melbourne, Victoria, 3010 Australia; 2 School of Geography, Earth and Atmospheric Sciences, The University of Melbourne, Victoria, 3010 Australia; 3 Elemental Scientific Lasers, LLC, 685 Old Buffalo Trail, Bozeman, MT 59715 USA; 4 Linus Pauling Institute and Department of Biochemistry and Biophysics, Oregon State University, Corvallis, OR 97331 USA; 5 Department of Anatomical Pathology, The Alfred Hospital, Victoria, 3004 Australia; 6 Mental Health Program, Department of Cell and Molecular Biology, Queensland Institute of Biomedical Research Berghofer, Herston, QLD 4006 Australia; 7 School of Chemistry and Bio21 Molecular Science and Biotechnology Institute, The University of Melbourne, Victoria, 3010 Australia; 8 Melbourne Dementia Research Centre, The University of Melbourne and Florey Institute of Neuroscience and Mental Health, Victoria, 3010 Australia; 9 Atomic Medicine Initiative, University of Technology Sydney, Ultimo, NSW 2007 Australia; 10 Department of Biochemistry, Emory University School of Medicine, Atlanta, GA 30322 USA

**Keywords:** Amyotrophic lateral sclerosis, Motor neuron disease

## Abstract

The copper compound Cu^II^(atsm) has progressed to phase 2/3 testing for treatment of the neurodegenerative disease amyotrophic lateral sclerosis (ALS). Cu^II^(atsm) is neuroprotective in mutant SOD1 mouse models of ALS where its activity is ascribed in part to improving availability of essential copper. However, SOD1 mutations cause only ~ 2% of ALS cases and therapeutic relevance of copper availability in sporadic ALS is unresolved. Herein we assessed spinal cord tissue from human cases of sporadic ALS for copper-related changes. We found that when compared to control cases the natural distribution of spinal cord copper was disrupted in sporadic ALS. A standout feature was decreased copper levels in the ventral grey matter, the primary anatomical site of neuronal loss in ALS. Altered expression of genes involved in copper handling indicated disrupted copper availability, and this was evident in decreased copper-dependent ferroxidase activity despite increased abundance of the ferroxidases ceruloplasmin and hephaestin. Mice expressing mutant SOD1 recapitulate salient features of ALS and the unsatiated requirement for copper in these mice is a biochemical target for Cu^II^(atsm). Our results from human spinal cord indicate a therapeutic mechanism of action for Cu^II^(atsm) involving copper availability may also be pertinent to sporadic cases of ALS.

## Introduction

The orally bioavailable and blood–brain barrier penetrant copper compound diacetyl-bis(4-methyl-thiosemicarbazonato)copper^II^ [Cu^II^(atsm)] is one of the most robustly tested and corroborated candidate drugs ever developed for the neurodegenerative condition of ALS, a fatal and rapidly progressive disease that selectively destroys motor neurons in the central nervous system (CNS). Pre-clinical studies involving mouse models of the disease show that oral treatment with Cu^II^(atsm) protects motor neurons in the spinal cord, mitigates the motor symptoms of neuronal decline, and extends survival^1–8^. These outcomes include efficacy when the treatment commences after symptom onset^2^. In animal model testing, Cu^II^(atsm) outperformed riluzole, the current first-line clinical prescription^2^. Cu^II^(atsm) is the first candidate drug for ALS to be independently validated by the ALS Therapy Development Institute^6^.

Copper is an essential micronutrient and its redox cycling between oxidised Cu^II^ and reduced Cu^I^ mediates catalytic activity in numerous vital cuproenzymes. The first discovered genetic cause of ALS was autosomal-dominant mutation of superoxide dismutase 1 (SOD1)^9^, a ubiquitously expressed antioxidant that requires copper for its catalytic activity^10^. Transgenic mice expressing mutant SOD1 are a robust animal model of ALS, possessing face and construct validity^11^. A consequence of this mutant SOD1 overexpression is insufficient copper availability, which is a CNS-specific defect despite ubiquitous expression of the disease-causing gene^12^. Expression of SOD1 in these models generates a pool of SOD1 that lacks copper in the catalytic site^1,3,5,12^, while the copper-dependent activities of cuproenzymes other than SOD1 are also affected^5,13^, indicating the causal gene has a broad impact on copper homeostasis. Treatment with Cu^II^(atsm) enables copper from the thiosemicarbazone complex to become incorporated into SOD1^3^, thereby converting aberrant copper-deficient SOD1 to its physiologically mature copper-replete form. These outcomes support a protective mechanism for Cu^II^(atsm) involving its ability to safely increase copper availability in the CNS.

Insufficient copper availability in mutant SOD1 mice is a CNS-specific defect despite ubiquitous expression of the disease-causing gene^12^. The copper-dependent activities of cuproenzymes other than SOD1 are also affected^5,13^, indicating the causal gene has a broad impact on copper homeostasis. However, SOD1 mutations account for only ~ 2% of ALS cases in the clinic, with most cases being sporadic and of unknown aetiology. Whether the efficacy of Cu^II^(atsm) in mutant SOD1 mice could translate to sporadic ALS through a mechanism of action involving copper availability, remains unclear. Notably however, treatment with Cu^II^(atsm) is neuroprotective in mouse models of neurodegeneration that do not involve the expression of mutant SOD1^7,14,15^. Nonetheless, to examine whether a protective mechanism of action related to copper availability may be relevant to human cases of sporadic ALS here we assessed human spinal cord samples from sporadic cases of the disease for levels of copper, cuproenzyme activity and the expression of regulatory pathways.

## Results

### Copper distribution is altered in human sporadic ALS affected spinal cord

**Figure 1:**
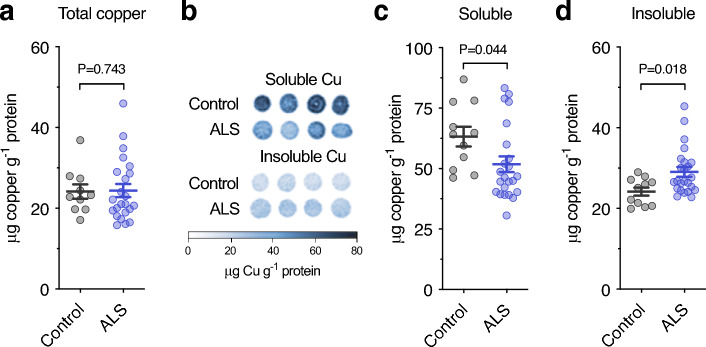
Copper in sporadic ALS-affected spinal cord relative to biochemical partitioning. (**a**) Copper in whole homogenate of human spinal cord prepared in tris-buffered saline (TBS) based extraction buffer and analysed using solution inductively coupled plasma-mass spectrometry (ICP-MS). (**b**) Representative microdroplet images illustrating use of laser ablation-ICP-MS (LA-ICP-MS) to measure copper levels in soluble and insoluble fractions of whole homogenates. **(c,d)** Copper in the TBS-soluble and TBS-insoluble fractions of human spinal cord homogenates measured using LA-ICP-MS. Symbols shown in **a**, **c** and **d** represent individual control and ALS cases and lines represent mean ± S.E.M. P values represent significance (t-test in **c**, Mann–Whitney test in **a** and **d**).

Total copper levels in human spinal cord samples were unaltered in sporadic cases of ALS (Fig. 1a**, **Table 1). However, analysis of TBS-soluble and TBS-insoluble fractions of spinal cord tissue via our laser ablation inductively coupled plasma mass spectrometry (LA-ICP-MS) microdroplet method^16^ revealed partitioning of endogenous copper in sporadic ALS was shifted towards the insoluble fraction (Fig. 1b–d). We examined whether ALS cases with the largest decrease in TBS-soluble copper also displayed the largest increase in TBS-insoluble copper, but this was not evident (Supplementary Fig. 1).

**Table 1:**
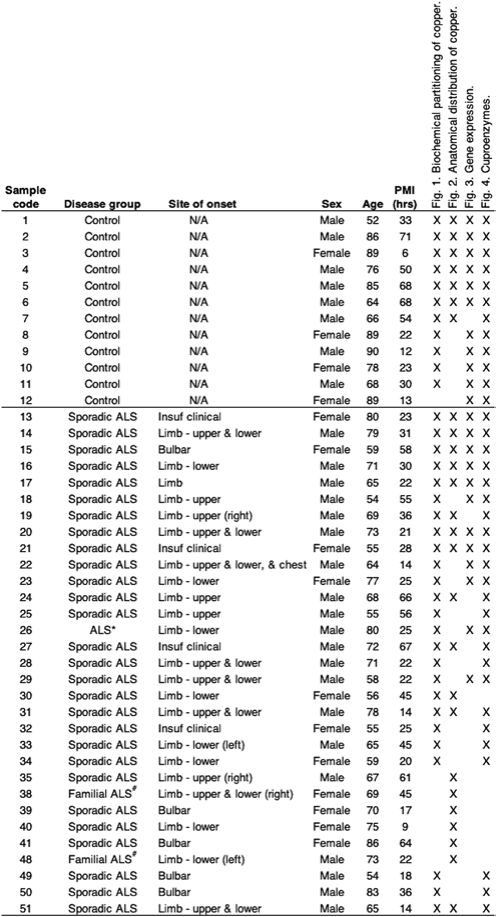
Sex, age and post-mortem interval details (± S.E.M.) for all study control and ALS cases.

PMI, post-mortem interval. Crosses denote the cases that were used in the various analyses.

*Adopted and no known genetic history of ALS; ^#^Mutation not known; Sporadic ALS, no known family history.

**Figure 2:**
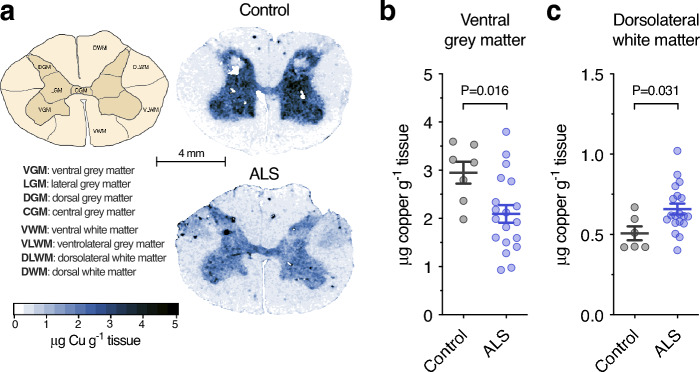
Copper in sporadic ALS-affected spinal cord relative to anatomical distribution. (**a**) Schematic illustration of anatomical regions of interest in transverse plane of human spinal cord sections and representative anatomical heatmaps for copper as measured using laser ablation-inductively coupled plasma-mass spectrometry (LA-ICP-MS). (**b**, **c**) Copper content in ventral grey matter and dorsolateral white matter regions of interest in human spinal cord measured using LA-ICP-MS. Results for additional regions of interest are shown in Table 2. Symbols shown in **b** and **c** represent individual control and ALS cases and lines represent mean ± S.E.M. P values represent significance (t-test in **b** and **c**).

In situ quantitation of copper by LA-ICP-MS^17–19^ revealed that in ALS endogenous copper was decreased in the grey matter and increased in the white matter, with the ventral horn grey matter and dorsolateral white matter regions being the principal anatomical sites of decrease and accumulation respectively (Fig. 2**, **Table 2). The ventral horn grey matter is the region within the spinal cord where the soma of motor neurons primarily reside and the corticospinal tracts traverse through the dorsolateral white matter column. These primary anatomical sites of pathology in ALS displayed the most conspicuous changes in copper (Fig. 2**, **Table 2).

**Table 2:**
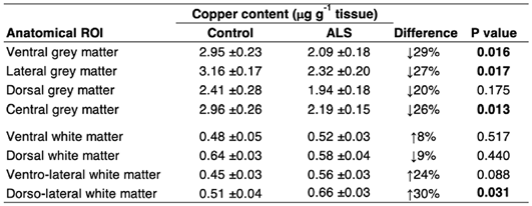
Copper content in anatomical sub-regions of human spinal cord.

Significant values are in [bold].

ROI, region of interest. Data presented as mean ± S.E.M. N = 6–7 controls, 17–19 ALS. *P* values represent significance (t-test for all measurements).

### Altered expression of copper handling genes in sporadic ALS

**Figure 3:**
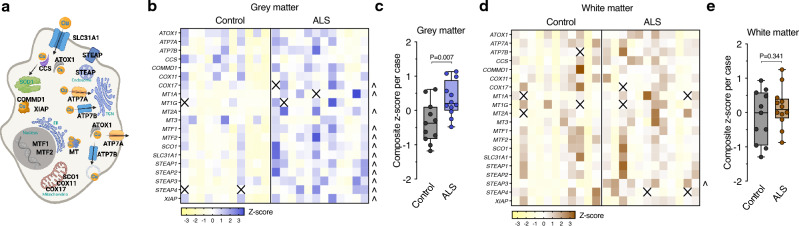
Molecular signature of copper imbalance in sporadic ALS. (**a**) Representation of gene products involved in the diverse mechanisms required for cellular copper handling. (**b**, **d**) Z-score heatmap showing expression of genes encoding copper transporters and chaperones in human spinal cord grey matter and white matter samples measured by quantitative RT-PCR. (**c,** **e**) Overall composite z-score for copper handling genes in individual control and ALS cases derived from results for grey matter and white matter regions as shown in heatmaps **b** and **d** respectively. Squares and symbols in panels (**b**-**d**) represent individual control and ALS cases. Box and whisker plots in **c** and **e** represent median, 25th and 75th percentiles, and min–max values. *P*-value in (**c**) represents significant difference between control and ALS cases. Arrowheads in (**b**) and (**d**) represent individual genes with increased expression in ALS and *P* < 0.05. (Mann–Whitney test for grey matter *MT1A*, *SLC31A1* and *STEAP2*, and for white matter *COX17* and *SCO1*. T-test for all other measurements.)

Physiological distribution of copper within cells involves co-ordinated function of a broad range of copper transporters and chaperones. Considering the prominent copper changes detected in spinal cord grey matter (Fig. 2**, **Table 2), we measured expression of 20 genes associated with cellular copper handling to gauge the extent to which these copper handling pathways may be affected in sporadic ALS. Reflecting the natural partitioning of copper to diverse cellular locations and cuproenzymes, this molecular machinery involves some discrete delivery pathways (*e.g.*, CCS mediated delivery of copper to SOD1) as well as some components that execute diverse roles (e.g., ATP7A involvement in protein processing at the trans-Golgi network or its involvement in cellular copper efflux). (Fig. 3a). Increased expression of 12 of the 20 genes analysed was detected in spinal cord grey matter from cases of sporadic ALS (Fig. 3b), providing an overall composite gene expression signature for altered copper homeostasis (Fig. 3c). Separate assessment of the same genes in spinal cord white matter revealed that although a similar overall trend was evident, the extent of changes in the spinal cord white matter was negligible when compared to the grey matter, with the expression of only one gene (*STEAP3*) significantly changed in the white matter (Fig. 3d,e). Gene expression results relative to symptom site of onset are shown (Supplementary Fig. 2).

### Cuproenzyme function is differentially altered in sporadic ALS affected spinal cord

**Figure 4:**
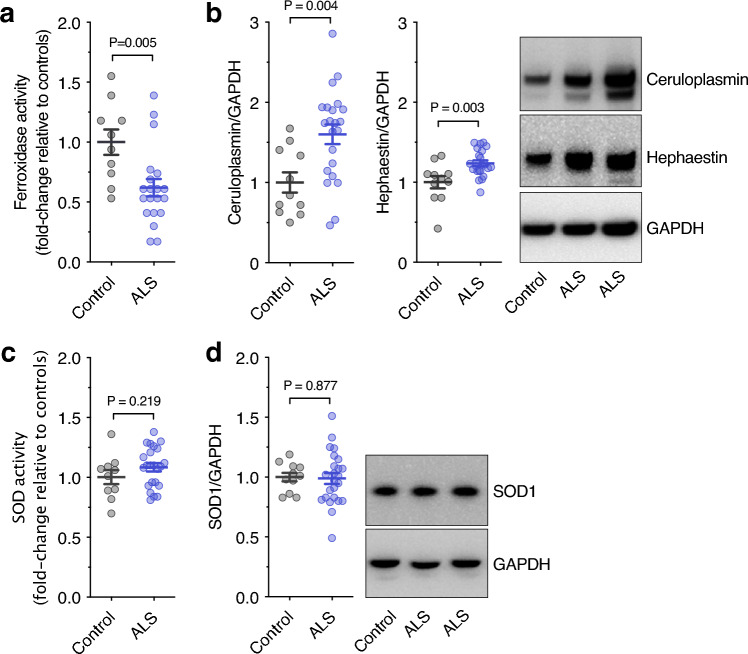
Abundance and activity of cuproenzymes in sporadic ALS. (**a**) Ferroxidase activity in human spinal cords measured as the rate of holo-transferrin produced min^-1^ mg^-1^ protein and expressed relative to control cases. (**b**) Ceruloplasmin and hephaestin protein levels in human spinal cord determined by western blot, normalised to the loading control GAPDH, and expressed relative to controls. (**c**) SOD activity in human spinal cords measured as inhibition of pyrogallol oxidation mg^-1^ protein and expressed relative to control cases. (**d**) SOD1 protein levels in human spinal cord determined by western blot, normalised to the loading control GAPDH, and expressed relative to controls. Representative western blot images are shown in (**b**) and (**d**). Symbols in graphs represent individual control and ALS cases and lines represent mean ± S.E.M. P values represent significance (t-test for all measurements). Full western blot images included in Supplementary Figs. 3 and 4.

SOD1 and ceruloplasmin both accumulate in copper-deficient states in spinal cords of mutant SOD1 mouse models of familial ALS^1,3,5,13^. Here, we assessed these cuproenzymes in human sporadic ALS-affected spinal cord. Ceruloplasmin and its homolog hephaestin promote cellular iron efflux via their multi-copper ferroxidase activity^20^. Total ferroxidase activity was decreased in sporadic ALS (Fig. 4a) even though ceruloplasmin and hephaestin protein levels were both increased (Fig. 4b; Supplementary Fig. 3). Consistent with mutant SOD1 mice^13^, this is indicative of an accumulation of these ferroxidases in a copper-deficient (inactive) state in sporadic ALS. In contrast to these ferroxidases, SOD1 protein levels and SOD1 activity were unchanged in sporadic ALS (Fig. 4c,d; Supplementary Fig. 4).

## Discussion

Although transgenic rodents expressing mutant SOD1 are the most robust animal model available for ALS, mutations affecting SOD1 account for only ~ 2% of all ALS cases, with ~ 90% of cases attributable to factors that are sporadic in nature and of no clear aetiology. It is notable, therefore, that therapeutic agents developed and tested in mutant SOD1 have a poor track record in translating to effective disease-modifying treatments for patients with sporadic ALS. While this has led some to question utility of the mutant SOD1 models for drug development, it has also prompted interrogation of the reproducibility of reported pre-clinical outcomes. To this end the ALS Therapy Development Institute has independently assessed prior reports of pre-clinical efficacy in mutant SOD1 mice and after assessing many other candidates the copper-containing compound Cu^II^(atsm) is the first putative treatment option that the ALS Therapy Development Institute has been able to validate^6^. This provides good support for Cu^II^(atsm). However, validation of pre-clinical studies involving mutant SOD1 models still does not address the issue of relevance to patients with a sporadic form of the disease.

Here, we provide results from human ALS-affected spinal cord that indicate a protective mechanism of action for Cu^II^(atsm) involving modulation of copper availability may link pre-clinical outcomes in mutant SOD1 mice to sporadic ALS. Evidence for disrupted copper availability in sporadic ALS is provided by our direct assessment of copper in human spinal cord. Conspicuously, these analyses revealed ventral grey matter, the primary anatomical site of pathology and neurodegeneration in ALS, as the spinal cord region with the greatest decrease in copper (Fig. 2**, **Table 2). Consistent with these changes impacting the physiological requirement for copper, altered expression was evident for the majority of genes involved in copper handling, resulting in an overall molecular signature for copper handling that is shifted in sporadic ALS (Fig. 3b,c). These gene expression changes did not highlight a specific copper related pathway (e.g., delivery of copper to SOD1). Rather, the range of genes affected indicated a more generalised change in cellular copper handling. But despite this generalised response, a relative absence of associated gene expression changes in spinal cord white matter from the same cases (Fig. 3d,e) indicates the biological impact of copper-related changes in sporadic ALS is restricted to the grey matter. This is consistent with neurodegeneration within the grey matter being the primary pathological basis of ALS. It is also consistent with copper changes in ALS being more pronounced in the grey matter than the white matter (Table 2).

We hypothesise that copper-related perturbations in sporadic ALS are not driven by a specific molecular change that has impacts restricted to a defined copper-related pathway. Rather, the gene expression changes reported here are more consistent with a biological response within grey matter in which diverse copper-related mechanisms are affected. This hypothesis is supported by some pre-clinical studies that have examined the protective activity of Cu^II^(atsm) in mutant SOD1 mice. Copper delivery to SOD1 is an understandable mechanism to examine in these mice due to the composition of the therapeutic agent and the biological requirement for copper in the ALS-related cuproenzyme. However, these prior studies have demonstrated that an unsatiated acquisition of copper by cuproenzymes in the spinal cord of mutant SOD1 mice is not restricted to SOD1 itself, indicating a perturbation to copper availability that has broad consequences. The most explicit evidence for this with respect to therapeutic significance came from a study in which mutant SOD1 mice were treated with Cu^II^(atsm) enriched with the natural copper isotope ^65^Cu. Administration of ^65^Cu^II^(atsm) followed by liquid chromatography-ICP-MS analysis confirmed that copper from the orally administered Cu^II^(atsm) entered the CNS pool of bioavailable copper, resulting in subsequent incorporation into multiple endogenous cuproproteins^3^ and improved activity of the cuproenzymes that accumulate in a copper-deficient state in mutant SOD1 mice^1,3,5,13^.

Here, we provide data which indicate the cuproenzymes ceruloplasmin and hephaestin both accumulate in a copper-deficient state in sporadic ALS (Fig. 4a,b). Although iron can be exported through other mechanisms, efflux via ferroportin requires oxidation of iron by ferroxidases and constitutes a major pathway in some cells. Thus, decreased copper-dependent ferroxidase activity could contribute to the accumulation of iron, which has been identified in ALS patients by magnetic resonance imaging^21^. An unsatiated requirement for copper in these cuproenzymes may therefore contribute to ALS. Notably, our analyses indicate copper perturbations in sporadic ALS cannot be broadly characterised as an overall copper insufficiency, indicating that ALS is not caused by simple tissue nutritional copper deficiency. This is apparent in our assessment of copper partitioning into TBS-soluble and -insoluble fractions of the spinal cord, with loss of copper from the soluble fraction and a concomitant increase in the insoluble fraction (**Fig. c,d**). It is possible that copper perturbations in ALS involve an internal redistribution that might benefit some cuproenzymes at the expense of others. This possibility is consistent with affinity gradients affecting the hierarchical order in which some cuproenzymes receive copper in preference to others^22^ and it is also consistent with the natural turnover of copper in the CNS being exceedingly slow compared to other organs and becoming even slower in conditions of limited copper bioavailability^23^. On a per patient basis, it may be possible that some ALS cases exhibit a copper perturbation that is more extensive when compared to other ALS cases, with the possibility, for example, that a high level of copper depletion in the soluble fraction equates to a high level of copper accumulation in the insoluble fraction. Cases examined in the present study do not reveal such an association. Further examination of additional features of copper-related processes may reveal additional insight and potentially support patient stratification for copper-based therapeutic interventions.

Cu^II^(atsm) labelled with radioactive isotopes of copper is utilised as a positron emission tomography (PET) imaging agent, with prominent use in imaging hypoxic tumour^24,25^. Other studies have explored its application to imaging diseases of the CNS^26,27^, including sporadic ALS where motor cortical accumulation of the PET signal correlates with symptomatic stage of disease progression determined using the ALS Functional Rating Scale (Revised)^28^. The specific factors that drive the observed localised accumulation in ALS are not yet confirmed, but redox imbalance involving a hyper-reductive state is implicated^29,30^. In brief, cellular retention of copper from bis(thiosemicarbazonato)-copper^II^ compounds such as Cu^II^(atsm) is dictated, in part, by intracellular reduction of the copper followed by its dissociation from the ligand. Electron donating methyl groups on the atsm ligand confer a reduction potential to Cu^II^(atsm) which means the copper is relatively resistant to intracellular reduction under physiological conditions. However, under hypoxic conditions electron flux through the mitochondrial electron transport chain is impeded, resulting in a hyper-reductive state that promotes reduction of the copper in Cu^II^(atsm) and its dissociation from the complex. Significantly, impaired electron flux through the electron transport chain even under normoxic conditions is sufficient to generate the hyper-reductive state that promotes reduction and dissociation of Cu^II^(atsm) within cells^29,30^. Mitochondrial dysfunction and/or tissue hypoxia in ALS^31^ may provide conditions within the disease-affected tissue that promote selective release of copper from Cu^II^(atsm). The presence of an aberrant pool of copper-deficient cuproenzymes within the same tissue will also promote localised accumulation of the copper through binding to cuproproteins which is a determinant of cellular retention of the copper^32^. The preferential release and retention of copper from Cu^II^(atsm) provides a plausible explanation for a PET signal in ALS patients that highlights the affected motor cortex^28^.

Additional mechanisms for the neuroprotective activity of Cu^II^(atsm) have been described. The compound exhibits potent anti-inflammatory effects^15,33^, it inhibits ferroptosis, an iron-dependent form of non-apoptotic cell death^34^, and it protects against peroxynitrite-driven toxicity^14^. All of these protective activities are pertinent to aberrant processes associated with ALS, including sporadic ALS, and therefore may also contribute to the potential therapeutic mechanism of Cu^II^(atsm) in treating ALS. Moreover, these potential therapeutic targets are also pertinent to other neurodegenerative diseases. Cu^II^(atsm) treatment results in neuroprotection and mitigation of symptoms of neuronal decline in four different animal models of Parkinson’s disease (PD)^14^, leading to phase 1 clinical assessment of the compound in PD patients^35^. And as in ALS patients, monitoring radio-labelled Cu^II^(atsm) in PD patients via PET imaging shows selective accumulation in the disease-affected basal ganglia^27^. As above, this selective accumulation implies region-specific influences of a hyper-reductive state in PD, a possibility consistent with evidence for mitochondrial dysfunction in PD^36^. It is also consistent with evidence for copper insufficiency in the PD-affected brain, including decreased copper levels, decreased ceruloplasmin ferroxidase activity, and the accumulation of copper-deficient SOD1^37–39^.

Cu^II^(atsm) produced favourable outcomes from an open-label phase 1 trial in which the ALS Functional Rating Scale (Revised), Edinburgh Cognitive and Behavioural ALS Screen, and seated forced vital capacity were included as secondary outcome measures^40,41^. This supported initiation of a blinded, placebo-controlled phase 2/3 trial^42^. A recent report on histological assessment of post-mortem tissue collected from patients enrolled in this study concluded there was no evidence for mitigation of neuropathology in the small number of cases examined (6 who received Cu^II^(atsm) compared to 6 who did not). This included a reported absence of any effect on neuronal density and an absence of any effect on TDP-43 pathology^43^. The former can be anticipated given that all cases had reached fatal disease end-stage. The latter involved an 82% decrease in TDP-43 pathology in the motor cortex and an 84% decrease in the spinal cord but neither of these reported TDP-43 pathology results were associated with statistical significance (*p* = 0.1 and *p* = 0.2 respectively) as the study was underpowered based on the reported error margins. Notably, a result indicative of microglial activation (Iba1 reactivity) was significantly decreased in sporadic ALS patients who were treated with Cu^II^(atsm) (63% decrease in spinal cord, *p* = 0.03)^43,44^ and this is consistent with a reported effect of Cu^II^(atsm) in mutant SOD1 mouse models of the disease^1,3,4^. Significantly, the density of activated microglia in ALS corticospinal tract has been found to positively correlate with the rate of ALS progression^45^.

Pre-clinical support for Cu^II^(atsm) in the treatment of ALS is primarily derived from in vivo studies involving mutant SOD1 animal models of the disease^1–7^, raising questions concerning applicability to the preponderant number of cases which does not involve mutant SOD1. Here, we show that disrupted copper availability is a feature of sporadic ALS, recapitulating features of the unsatiated requirement for copper that is evident in mutant SOD1 mice. Preliminary histology results from cases of sporadic ALS who were treated with Cu^II^(atsm) provide some evidence for translatability of outcomes from mutant SOD1 mice to the human sporadic disease^1,3,4,43^, and when used as a PET tracer, Cu^II^(atsm) selectively accumulates in the affected motor cortex of sporadic ALS patients. These multiple lines of evidence indicate the therapeutic benefits of Cu^II^(atsm) treatment derived from pre-clinical studies utilising mutant SOD1 mice may be pertinent to sporadic cases of the disease.

## Methods

### Human tissue and processing

Procedures involving post-mortem human tissue were approved by a University of Melbourne Human Ethics Committee (Project ID 1238124) and were performed in accordance with National Health and Medical Research Council guidelines and regulations. Frozen sections of lumbar spinal cord were obtained from the Victorian Brain Bank (Australia) and the MS Society Tissue Bank (UK) where samples had been donated with informed consent. All ALS cases had reached clinical end-stage. Tissue used in microdroplet and biochemical analyses was processed to generate tris(hydroxymethyl)aminomethane-buffered saline (TBS)-soluble and -insoluble fractions as previously described^13^. Assays involving SOD1 utilised TBS soluble extracts. Assays involving ceruloplasmin and hephaestin utilised TBS-insoluble fractions that were supplemented with 1% (v/v) triton X-100 then centrifuged (18,000 RCF, 4 °C, 5 min) to produce triton X-100 soluble extracts. Grey and white matter material dissected from spinal cord samples was processed for gene expression as described below. Table 1 provides details for all cases used in the study and shows which cases were used for the various assays. The amount of spinal cord tissue available precluded subjecting all cases to all analyses.

### Copper quantitation

Total copper levels in human spinal cord were measured by solution inductively coupled plasma-mass spectrometry (ICP-MS) as previously described^1^ using an Agilent 7700 Series ICP-MS. To assess biochemical partitioning of copper, TBS-soluble and TBS-insoluble fractions were analysed using a microdroplet laser ablation (LA) ICP-MS methodology^16^. Parallel assessment of protein content via the BCA Assay (Thermo Fisher Scientific) enabled expression of copper content in total spinal cord and the TBS-soluble and -insoluble fractions relative to protein content. In situ quantitation of copper was performed using LA-ICP-MS^18^ utilising spinal cord embedded in Optimal Cutting Temperature compound and cryo-sectioned at 30 μm in the transverse plane. Reference to a CNS matrix-matched standard^46^ enabled expression of in situ copper relative to tissue mass. All in situ and microdroplet LA-ICP-MS analyses utilised a NewWave Research NWR213 laser ablation system coupled to an Agilent 8800 triple quadrupole ICP-MS. Data were analysed using Iolite operating under the Igor Pro 8 suite (WaveMetrics, Inc.).

### Gene expression

Frozen spinal cord samples were dissected macroscopically to separate distinct grey and white matter regions. Weighed samples of grey and white matter (~ 10 mg) were then prepared for gene expression analyses using manufacturer’s instructions as follows: Nucleic acids were extracted (TRI-Reagent, Sigma); contaminating gDNA in isolated mRNA was degraded (Turbo DNA-Free Kit, Thermo Fisher Scientific); mRNA quantity was measured (Qubit RNA HS Assay Kit, Thermo Fisher Scientific); cDNA was synthesised (High Capacity cDNA Reverse Transcription Kit, Thermo Fisher Scientific); and cDNA (25 ng) was pre-amplified for all genes assessed (Taqman PreAmp Master Mix and Taqman Gene Expression Assays (Supplementary Table 1), Thermo Fisher Scientific). Pre-amplified cDNA was diluted 20-fold, then quantitative RT-PCR performed (Taqman Gene Expression Assays and Taqman Fast Advanced Mastermix, Thermo Fisher Scientific) on samples in triplicate using a QuantStudio 6 Flex system (Thermo Fisher Scientific). Relative expression of individual genes involved in cellular copper handling was determined via the ΔΔct method normalised to *GAPDH*. Composite z-score values representing changes affecting overall copper handling were calculated for each control or ALS case as the average z-score across all genes analysed.

### SDS-PAGE and immunoblotting

Proteins were resolved by SDS-PAGE and assessed by immunoblotting using methods previously described^13^. Primary antibodies used were raised to detect: SOD1 (Abcam, ab79390); GAPDH (Cell Signaling Technology, 2118); ceruloplasmin (DAKO, Q0121); and hephaestin (Santa Cruz, sc365365). Detection utilised horseradish peroxidase conjugated secondary antibodies for anti-rabbit IgG (Cell Signaling Technology, 7074) or anti-mouse IgG (Cell Signaling Technology, 7076) followed by enhanced chemiluminescence (ECL Advance, GE Healthcare). Abundance of cuproenzymes of interest was normalised to the loading control GAPDH and expressed relative to control cases.

### Cuproenzyme activity

Ferroxidase activity in triton X-100 extracts and SOD1 activity in TBS-soluble extracts were determined following previously described procedures^13^. In brief, ferroxidase activity involved assessment of holo-diferric transferrin production using human apo-transferrin as a substrate and supplying iron in the form of FeSO_4_^47,48^. SOD1 activity involved assessment of inhibition of pyrogallol oxidation^49^. Both activities are presented as fold-change relative to control cases.

### Statistical analyses

All statistical analyses were performed using GraphPad Prism. Data sets were assessed for statistical outliers using the ROUT method^50^ and for Gaussian distribution. Data are presented as mean ± S.E.M. except for transcript data which are presented as z-scores. Significant differences between groups were determined using two-tailed t-tests when data fit Gaussian distribution parameters or the Mann–Whitney test when data did not fit Gaussian distribution parameters. Significance was determined as *p* < 0.05.

### Supplementary Information

Supplementary Information.

## Data Availability

All data supporting the conclusions of this article are included within the article.
