# Supplementary material for: Evidence for disrupted copper availability in human spinal cord supports CuII(atsm) as a treatment option for sporadic cases of ALS
Source: Sci Rep. 2024 Mar 11;14:5929. doi: 10.1038/s41598-024-55832-w (PMC10928073; doi:10.1038/s41598-024-55832-w)
Supplement: Supplementary file 1 — Supplementary Information. [file 41598_2024_55832_MOESM1_ESM.pdf]

Supplementary Information: Evidence for disrupted copper availability in human spinal cord supports Cu^{II}(atsm) as a treatment option for sporadic cases of ALS

James BW Hilton[1], Kai Kysenius[1], Jeffrey R Liddell[1], Stephen W. Mercer[1], Bence Paul[2,3], Joseph S Beckman[4], Catriona A McLean[5], Anthony R White[6], Paul S Donnelly[7], Ashley I Bush[8], Dominic J Hare[9], Blaine R Roberts[10], Peter J Crouch[1,*]

[1]Department of Anatomy & Physiology, The University of Melbourne, Victoria 3010, Australia
[2]School of Geography, Earth and Atmospheric Sciences, The University of Melbourne, Victoria 3010, Australia.
[3]Elemental Scientific Lasers, LLC. 685 Old Buffalo Trail, Bozeman, MT 59715, United States.
[4]Linus Pauling Institute and Department of Biochemistry and Biophysics, Oregon State University, OR 97331, United States
[5]Department of Anatomical Pathology, The Alfred Hospital, Victoria 3004, Australia
[6]Mental Health Program, Department of Cell and Molecular Biology, Queensland Institute of Biomedical Research Berghofer, Queensland 4006, Australia
[7]School of Chemistry and Bio21 Molecular Science and Biotechnology Institute, The University of Melbourne, Victoria 3010, Australia
[8]Melbourne Dementia Research Centre, The University of Melbourne and Florey Institute of Neuroscience and Mental Health, Victoria 3010, Australia
[9]Atomic Medicine Initiative, University of Technology Sydney, New South Wales 2007, Australia.
[10]Department of Biochemistry, Emory University School of Medicine, GA 30322, United States.
*Corresponding author: Peter Crouch, Department of Anatomy & Physiology, The University of Melbourne, Victoria 3010, Australia. Phone: +61 3 8344 4292 E-mail: pjcrouch@unimelb.edu.au

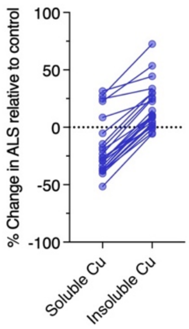

Supplementary Fig. 1. Case-by-case comparison of changes in TBS-soluble and TBS-insoluble copper in ALS . Data show change in TBS-soluble and TBS-insoluble copper in ALS cases as a percentage of control cases. Individual cases for both copper measurements are indicated via connecting lines. Data are derived from results presented in Fig. 1c and 1d.

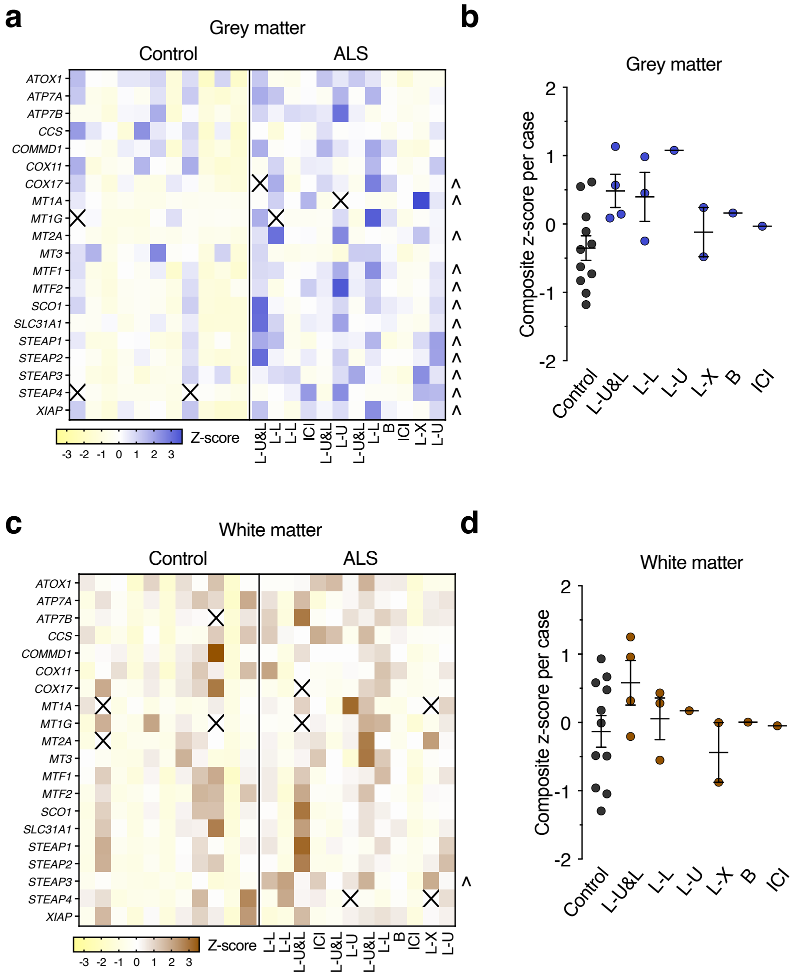

Supplementary Fig. 2. Molecular signature of copper imbalance in sporadic ALS relative to symptom site of onset. **(a,c)** Z-score heatmap showing expression of genes encoding copper transporters and chaperones in human spinal cord grey matter and white matter samples measured by quantitative RT-PCR. **(b,d)** Overall composite z-score for copper handling genes in individual control and ALS cases derived from results for grey matter and white matter regions as shown in heatmaps **a** and **b** respectively. Squares and symbols in all panels represent individual control and ALS cases. Site of onset for ALS cases segregated as follows: L-U&L, limb onset - upper and lower limbs; L-L, limb onset - lower limbs; L-U, limb onset - upper limbs; L-X, limb onset - undefined; B, bulbar onset; ICI, insufficient clinical information.

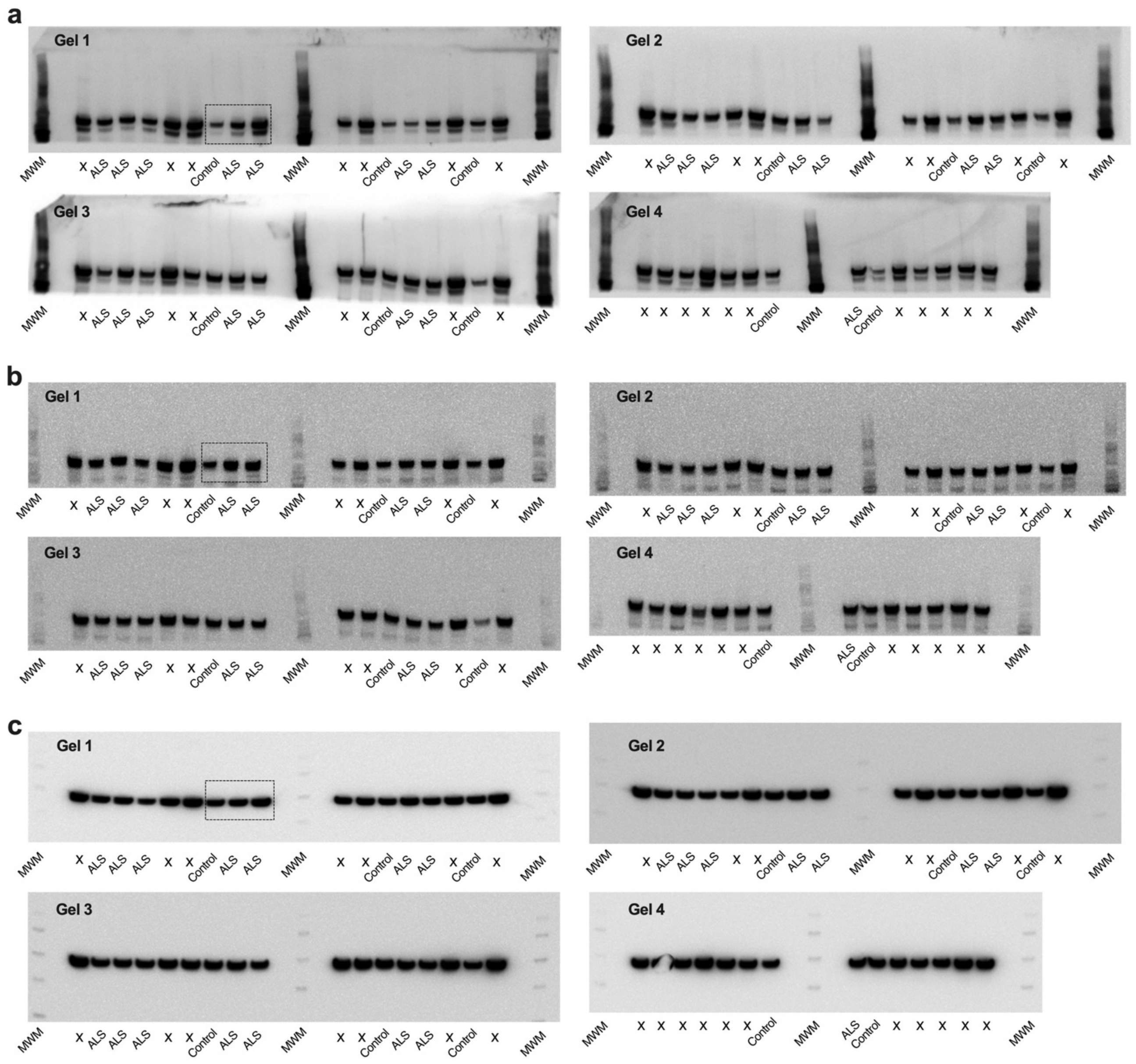

Supplementary Fig. 3. Full western blot images for ceruloplasmin and hephaestin in human spinal cord. (a) Ceruloplasmin and **(b)** hephaestin levels in samples loaded onto four separate gels. Images for each protein were derived from the same membranes. **(c)** Corresponding levels of the loading control GAPDH. For all gels, each lane represents a sample derived from a unique ALS case or control. Crosses represent spinal cord extracts from human cases that were neither ALS nor control and were not assessed as part of the current study. "MWM" represents molecular weight marker. Dashed boxes highlight the regions used for representative images included in Fig. 4b.

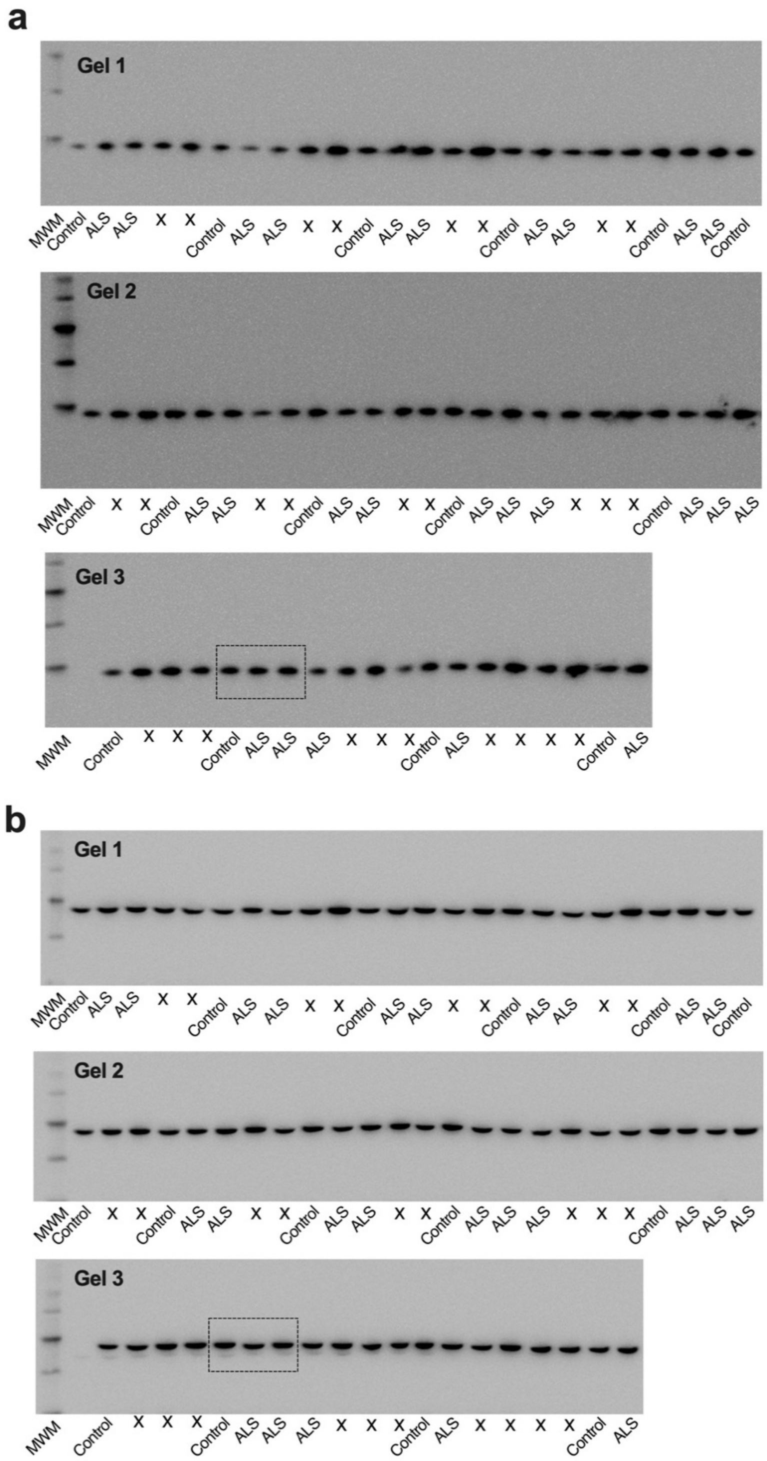

Supplementary Fig. 4. Full western blot images for SOD1 in human spinal cord. (a) SOD1 levels in samples loaded onto three separate gels. **(b)** Corresponding levels of the loading control GAPDH. For all gels, each lane represents a sample derived from a unique ALS case or control. Crosses represent spinal cord extracts from human cases that were neither ALS nor control and were not assessed as part of the current study. “MWM” represents molecular weight marker. Dashed boxes highlight the regions used for representative images included in Fig. 4d.

Supplementary Table 1. TaqMan Gene Expression Assays used for gene expression analyses. Gene name, protein name, and corresponding TaqMan Assay ID# for analysing expression of copper handling genes in human tissue. *GAPDH* was used for expression normalisation.

Gene	Protein	TaqMan Assay ID#
ATOX1	Antioxidant 1 copper chaperone	Hs00187841_m1
ATP7A	ATPase copper transporting Alpha	Hs00163707_m1
ATP7B	ATPase copper transporting Beta	Hs01075310_m1
CCS	Copper chaperone for superoxide dismutase	Hs00192851_m1
COMMD1	Copper metabolism domain containing 1	Hs00415059_m1
COX11	Cytochrome c oxidase copper chaperone COX11	Hs00362087_m1
COX17	Cytochrome c oxidase copper chaperone COX17	Hs01053235_g1
MT1A	Metallothionein 1A	Hs00831826_s1
MT1G	Metallothionein 1G	Hs04401199_s1
MT2A	Metallothionein 2A	Hs02379661_g1
MT3	Metallothionein 3	Hs00359394_g1
MTF1	Metal regulatory transcription factor 1	Hs00232306_m1
MTF2	Metal regulatory transcription factor 2	Hs00980938_m1
SCO1	Cytochrome c oxidase assembly protein	Hs01552201_m1
SLC31A1	Solute carrier family 31 member 1	Hs00977266_g1
STEAP1	Six transmembrane epithelial antigen of the prostate 1	Hs00185180_m1
STEAP2	Six transmembrane epithelial antigen of the prostate 2	Hs00401292_m1
STEAP3	Six transmembrane epithelial antigen of the Prostate 3	Hs00217292_m1
STEAP4	Six transmembrane epithelial antigen of the prostate 4	Hs01026584_m1
XIAP	X-linked inhibitor of apoptosis	Hs00745222_s1
GAPDH	Glyceraldehyde-3-phosphate dehydrogenase	Hs02758991_g1
